# Correction to: MicroRNA-30d promotes angiogenesis and tumor growth via MYPT1/c-JUN/VEGFA pathway and predicts aggressive outcome in prostate cancer

**DOI:** 10.1186/s12943-019-1051-x

**Published:** 2019-08-06

**Authors:** Zhuo-yuan Lin, Guo Chen, Yan-qiong Zhang, Hui-chan He, Yu-xiang Liang, Jian-heng Ye, Ying-ke Liang, Ru-jun Mo, Jian-ming Lu, Yang-jia Zhuo, Yu Zheng, Fu-neng Jiang, Zhao-dong Han, Shu-lin Wu, Wei-de Zhong, Chin-Lee Wu

**Affiliations:** 1 Department of Urology, Guangdong Key Laboratory of Clinical Molecular Medicine and Diagnostics, Guangzhou First People’s Hospital, Guangzhou Medical University, Guangzhou, 510180 China; 2 Department of Urology, The Second Affiliated Hospital of Guangzhou Medical University, Guangzhou Medical University, Guangzhou, 510260 China; 3 Institute of Chinese Materia Medica, China Academy of Chinese Medical Sciences, Beijing, 100700 China; 4 Guangdong Provincial Institute of Nephrology, Nanfang Hospital, Southern Medical University, Guangzhou, 510515 China; 5 Urology Key Laboratory of Guangdong Province, The First Affiliated Hospital of Guangzhou Medical University, Guangzhou Medical University, Guangzhou, 510230 China; 6 Graduate school of Jinan University, Guangzhou, 510632 China; 7 Department of Pathology, Massachusetts General Hospital and Harvard Medical School, Boston, MA 02114 USA; 8 Department of Urology, Massachusetts General Hospital and Harvard Medical School, Boston, MA 02114 USA

**Correction to: Mol Cancer (2017) 16:48**

**https://doi.org/10.1186/s12943-017-0615-x**

After publication of the article [1], the author reported that this article contained some errors.

The photograph of sh-NC in Fig. 1c was misplaced. The correct version of the figure and the figure legend is presented below.

**Fig. 1:**
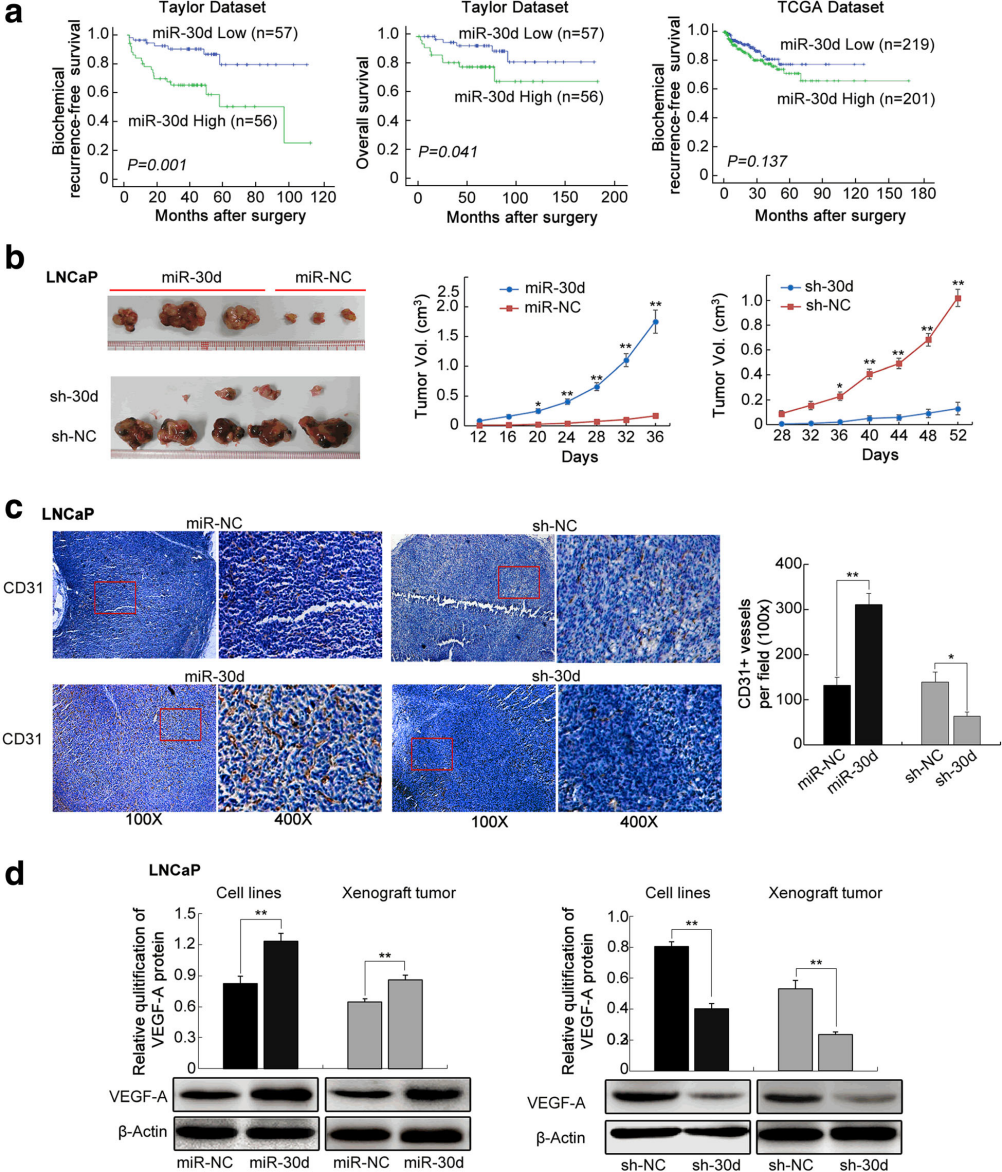
Prognostic value of miR-30d expression in PCa patients and its functions on tumor growth and angiogenesis in vivo using LNCaP cell induced tumor xenografts. **a** Kaplan-Meier analyses of biochemical recurrence (BCR)-free survival and overall survival of PCa patients based on miR- 30d expression in Taylor and TCGA datasets. **b** LNCaP cells stably expressing miR-30d formed significantly larger tumor nodules and remarkably speeded up tumor xenografts growth compared with the controls. Conversely, PCa cells that permanently suppressed miR-30d expression led to the smaller tumor nodules and the slower tumor growth compared with the control. **c** Immunohistochemical analysis using pan-endothelial marker CD31 antibody. **d** VEGFA protein expression in different groups detected by Western blot analysis. Data were presented as Mean ± SD. *P < 0.05. **P < 0.01

## References

[1] Lin Z-y, Chen G, Zhang Y-q, He H-c, Liang Y-x, Ye J-h, Liang Y-k, Mo R-j, Jian-ming L, Zhuo Y-j, Zheng Y, Jiang F-n, Han Z-d, Shu-lin W, Zhong W-d, Chin-Lee W (2017). MicroRNA-30d promotes angiogenesis and tumor growth via MYPT1/c-JUN/VEGFA pathway and predicts aggressive outcome in prostate cancer. Mol Cancer.

